# Supplementary figures and images for: PD-1 Immune Checkpoint Blockade Promotes Therapeutic Cancer Vaccine to Eradicate Lung Cancer
Source: Vaccines (Basel). 2020 Jun 18;8(2):317. doi: 10.3390/vaccines8020317 (PMC7350208; doi:10.3390/vaccines8020317)

Figure S1

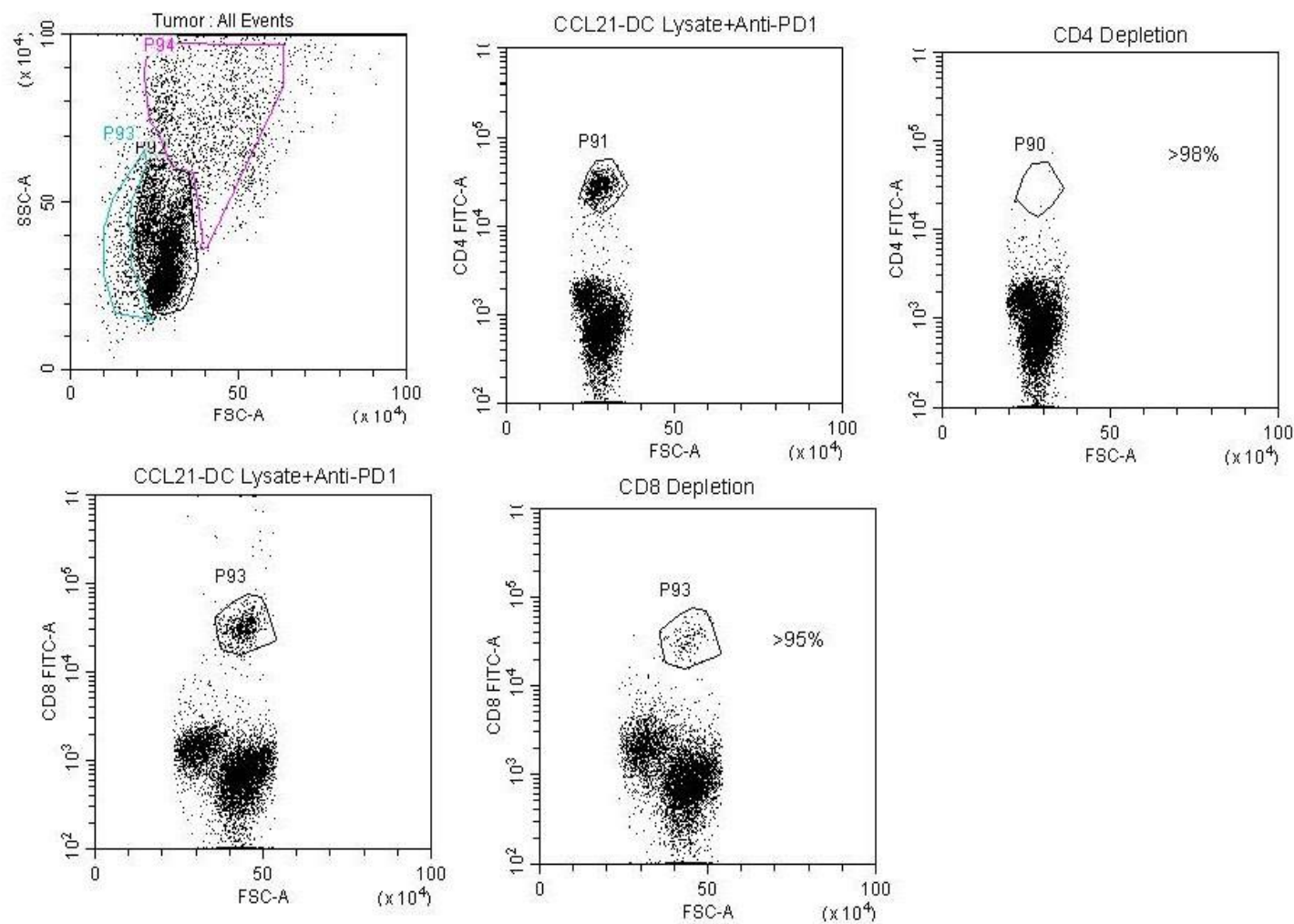

**Figure S1.** CD4 and CD8 T cell depletion.

Supplement: Supplementary file 1 [file vaccines-08-00317-s001.pdf]
